# Spider diversity (Arachnida: Araneae) in Atlantic Forest areas at Pedra Branca State Park, Rio de Janeiro, Brazil

**DOI:** 10.3897/BDJ.4.e7055

**Published:** 2016-01-15

**Authors:** Pedro Castanheira, Abel Pérez-González, Renner L. C. Baptista

**Affiliations:** ‡ Universidade Federal do Rio de Janeiro, Rio de Janeiro, Brazil; § Museo Argentino de Ciencias Naturales "Bernardino Rivadavia", Buenos Aires, Argentina

**Keywords:** Survey, faunistics, Neotropics, South America

## Abstract

**Background:**

There has never been any published work about the diversity of spiders in the city of Rio de Janeiro using analytical tools to measure diversity. The only available records for spider communities in nearby areas indicate 308 species in the National Park of Tijuca and 159 species in Marapendi Municipal Park. These numbers are based on a rapid survey and on an one-year survey respectively.

**New information:**

This study provides a more thorough understanding of how the spider species are distributed at Pedra Branca State Park. We report a total of 14,626 spider specimens recorded from this park, representing 49 families and 373 species or morphospecies, including at least 73 undescribed species. Also, the distribution range of 45 species was expanded, and species accumulation curves estimate that there is a minimum of 388 (Bootstrap) and a maximum of 468 species (Jackknife2) for the sampled areas. These estimates indicates that the spider diversity may be higher than observed.

## Introduction

The Atlantic Forest is one of the largest centers of biodiversity in the world (Myers et al. 2000). Its original area covered around 15% of the Brazilian territory, from the state of Rio Grande do Sul to the state of Piauí, and also portions of Paraguay and Argentina. However, only around 8% of its original cover remains preserved (Fundação SOS Mata Atlântica / Instituto Nacional de Pesquisas Espaciais 2011). Many of the remaining fragments are under intense anthropic pressure and are unevenly distributed throughout the biome, which hinders the preservation of endemic and threatened species (MMA: Ministério do Meio Ambiente 2012).

A large portion of Rio de Janeiro city is covered by remnants of the Atlantic Forest, distributed on three large mountain ranges: Gericinó-Mendanha to the north, Tijuca to the southeast, and Pedra Branca to the southwest. The latter includes the Pedra Branca State Park, or Parque Estadual da Pedra Branca (ICMBIO 2008).

Despite considerable abundance in the Atlantic Forest, arthropod communities in the area are still poorly known. Terrestrial arthropods represent the largest proportion of the known biotic diversity in the world, where many highly diverse arthropod taxa are excellent bioindicators, even at small scale (Andersen 1990, Schowalter 1995, Brown 1997, Fisher 2000, Ferrier et al. 2004).

Spiders (Araneae) and other arachnids present high diversity, high abundance, and variable life styles. They are one of the main predators in terrestrial environments with a considerable impact upon prey population, acting as agents of biological control (Nyffeler and Benz 1987, Riechert and Lockley 1984, Young and Edwards 1990). Spiders, in particular, are highly diverse, with more than 45,000 species in 114 families (World Spider Catalog 2015). According to Agnarsson et al. (2013), around 50 % of the deposited material in collections around the planet is composed by undescribed species. Some estimates point out that more than 80,000 species of spiders still await description (Brescovit 1999).

Spiders are a choice group for the carrying out species surveys with standardized techniques, because they are abundant and easily found (Coddington et al. 1991, Cardoso et al. 2008). Fieldwork may then be composed by active and passive sampling techniques during specific unit of time, allowing for the comparison of species diversity and study of the structure of the community of spiders (Coddington et al. 1991, Scharff et al. 2003).

Neotropical spider fauna seems to be the least known among the high diversity areas around the globe. One example of our incipient knowledge is the citation in Brescovit et al. (2011) of only 3,203 spider species for Brazil, included in 72 of the 112 known families at that time. However, this number is clearly underestimated, as the real diversity surely is much higher. Until now, there are records about the richness of species of spiders for some Brazilian states, like Amazonas (e.g. Nogueira et al. 2014), Bahia (e. g. Melo et al. 2014), Mato Grosso (e.g. Raizer et al. 2005), São Paulo (e. g. Candiani et al. 2005, Indicatti et al. 2005, Brescovit et al. 2011) and Rio Grande do Sul (e. g. Buckup et al. 2010). Additionally, there is a recent book chapter about the spider fauna of Parque Municipal do Marapendi in the city of Rio de Janeiro (Baptista et al. 2015) and other unpublished data (Santos et al. 2009).

According to Brescovit et al. (2011), the known spider fauna for São Paulo state is represented by 875 described species, in 50 families. There are also records of 808 described species in 51 families for Rio Grande do Sul (Buckup et al. 2010). In comparison, our knowledge on the Rio de Janeiro spider fauna includes 953 described species, as well as roughly 287 undescribed species and 258 morphospecies. According to the monograph of Quintarelli (2014) and a database compiled by R. Baptista (UFRJ) and partners, there are 1,498 species or morphospecies recorded for Rio de Janeiro in 61 families. Despite its small area, the spider fauna from Rio de Janeiro is the most diverse and best known in comparison to other states in Brazil according to available data. However, the current species list for Rio de Janeiro is still limited, incomplete and has never been published.

This paper presents a standardized survey of the spider fauna and statistical estimates of the diversity in four forested areas at Pedra Branca State Park, the largest urban forest of Rio de Janeiro city. Previously, there were only records of sporadic fieldwork and rare citations in the literature about the spiders from the park, including type material of two species and specimens belonging to only 26 additional species or morphospecies.

## Material and methods

### Study Area

**Figure 1.**
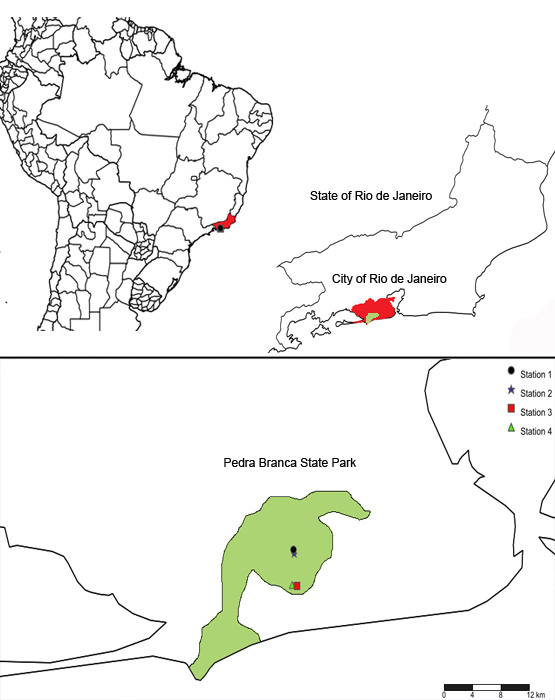
Collection stations in Pedra Branca State Park.

**Figure 2.**
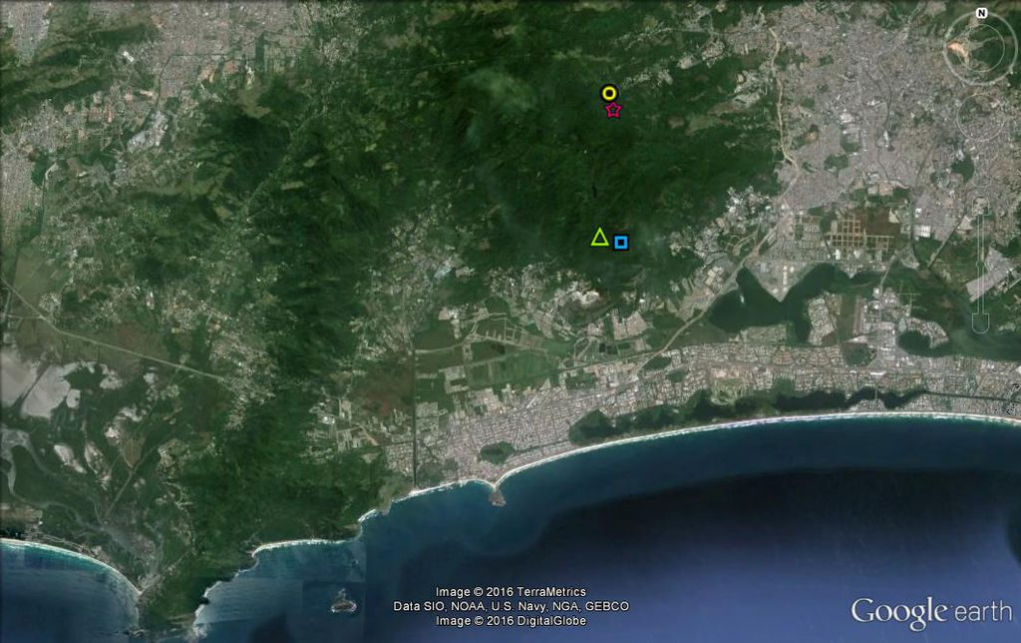
Pedra Branca State Park on Google Earth. Circle: Station 1; Star: Station 2; Square: Station 3 and Triangle: Station 4.

The Pedra Branca range is located at the municipality of Rio de Janeiro, between 22º 55’ - 23º 05’ S and 43º 20’ - 43º 40’ W (Figs 1, 2). It has 197.27 Km² of area, aproximately 12,500 ha. The area of the park includes all slopes above 100 meters and forested areas nearby, surrounded by plain areas already occupied by urban sprawl (Coura et al. 2009). It is estimated that 6,920 ha (55% of the area) is covered by well-preserved forest and that 3,216 ha (26%) is under strong anthropic pressure, now covered by regenerating forests and grass fields. The Pedra Branca State Park was created in 1974 in order to protect the remaining natural environment, and especially the hydrographic network (Coura et al. 2009).

Four work stations were chosen for the present study. Each one is considered as the center of a square of 100 m^2^, in which the spiders were collected. In the park, there are only three access points, consisting therefore of three core stations. One of them is distant and the area is covered by recent vegetation (Piraquara core) whereas the other two (Pau-da-Fome and Camorim cores) have trails penetrating a more protected, older forest. Thus, two work stations were located near Pau-da-Fome (the main core station of the park) and two nearby the Camorim accessory core. The stations were chosen due to their preservation state and security reasons. One station in each access point was chosen in the best preserved areas we could find. The other stations were chosen near heavily used trails and considered as under higher anthropic effect with clear evidence of recent succession. The stations are:

**Station 1 (S1)**: Pau-da-Fome – Figueira (anthropic)

Station 1 is located at an area of trails nearby the headquarters of Pau-da-Fome core station and the river that crosses the region. This area is subjected to a high influx of park visitors due to its easy access and use of the river for recreation. Here, the trees are smaller and more widely spaced with high abundance of bushes, soil bromeliads and exotic plants, as the big fig tree after which the trail is named.

Location: 22°55'57.0"S and 043°26'32.3"W. Elevation: 138 m.

**Station 2 (S2)**: Pau-da-Fome – Padaria

Station 2 was initially considered by us as one of the oldest forest areas at Pau-da-Fome. It is reached after a 20 minute walk in a secondary trail following one of the park streams. This area is not easily accessed by the public and comprises the ruins of an old farm from the 19th century, which is surrounded by moderately steep ravines. There are few exotic plants and bushes at this station, with a clearing in the ruins, where there are small trees and some bushes and herbs, alongside moderately closed canopy in the surroundings. The stream borders were covered by more bushes and herbs than the remaining points.

Location: 22°56'12.8"S and 043°26'29.1"W. Elevation: 133 m.

**Station 3 (S3)**: Camorim – Sede (anthropic)

This station is located just behind the accessory core of Camorim. This area is under high pressure due to the park facilities and the constant human activity for recreation and water use. Also, there is a dam in the Camorim river and equipment for collection and treatment of water for human consumption on the nearby areas of Rio de Janeiro city. The trees are smaller than in other stations and there is a large number of bushes and exotic trees. All the spider collections were done alongside the river banks and on the nearby ravines.

Location: 22°58'12.0"S and 043°26'16.4"W. Elevation: 160 m.

**Station 4 (S4)**: Camorim – Açude

This station is reached after a 45 minute walk in a steep trail that leads to a relatively large dike built for water collection. It is considered the best preserved area in this work. A larger number of higher trees and a denser canopy is found at this station, coupled with a small number of bushes and herbs, which indicate an older forest tract. There is no water course in the vicinity, but the river is located around 200 m from the station center, at the bottom of steep ravines.

Location: 22°58'08.3"S and 043°26'38.5"W. Elevation: 342 m.

### Collection Techniques and Identification of Specimens

Spiders were collected using methods adapted from the ones broadly used in similar studies (e. g. Coddington et al. 1991, Toti et al. 2000, Soerensen et al. 2002, Scharff et al. 2003, Cardoso et al. 2008). All active searching samples took 30 minutes each. The first method is called “looking up”, where all spiders seen from the knee to the highest point the researcher can reach were collected. This technique was done with one sample during the day (from 08:00 to 12:00) or in the afternoon (13:00 to 18:00), depending on field conditions, and two samples of 30 minutes each during the night (from 20:00 to 23:00). The second method called “looking down” aims to collect spiders found from the height of the knee to the ground. As in the previous technique, only one sample was done during the day, whereas two samples were made during the night. “Looking up” and “looking down” were the only active techniques used during nocturnal sampling. The third technique is called “sweeping”, where an entomologic net (diameter approximatelly 40 cm) was moved over herbs and bushes to collect spiders, up to the level of the researcher's knee. The fourth technique is called “beating”. This method allows the collection of spiders by shaking high vegetation while holding a 1 m² tray under it. The fifth technique is called “sieving”, aiming to collect little spiders that live in the litter, by sifting it on a 1 m² tray. The sixth technique is called “cryptic”, which is an active method to check on protected and hidden habitats, such as fallen tree trunks, tree and stone cracks and caves, in order to collect spiders that live in such conditions. Besides these active techniques, we used pitfall traps as the only passive technique. Twenty pitfall traps were buried in the ground in each sample station, during 8 days. Each trap was partially filled with supersaturated saline solution. All the spiders collected during this expedition were fixed in ethanol 75%.

The material was sorted into morphospecies at first. In contrast to many published surveys where the juveniles are discarded because they usually do not present many somatic characteristics to place them into morphospecies at species level, most juveniles were taken into account. Juveniles usually represent more than half of all collected specimens and its plain disposal implies in discarding a lot of useful information. The process of identification was conducted by comparison to published papers, type material, whenever possible, and online catalogs (e. g. World Spider Catalog 2015). All identifications were carried out by the authors and voucher specimens are deposited in the collection of the Laboratório de Diversidade de Aracnídeos/UFRJ. The voucher specimens collected during the survey are deposited under sample numbers PBR 001-5,889.

### Data Analyses

In this paper, parameters related to alpha diversity were evaluated by estimates of species richness using methods of accumulation curves (Clench equation) and non-parametric estimators: ICE, ACE, Chao1, Chao2, Jackknife 1, Jackknife 2 and Bootstrap. Richness estimates were possible with the use of the software EstimateS Richness Estimator Program, Version 9.1 (Colwell 1999). It generated estimates of species richness based on empirical data.The structure of the communities of spiders was also evaluated with index widely used in taxonomic surveys like: diversity of species with Shannon-Wiener (H’) Index, equitability with Pielou (J) Index and dominance by Berger-Parker Index (d), calculated by the usual mathematic formulas.

## Results

Adding up all information sources, 14,735 spider specimens were recorded for the park, including records from literature, museum collections and our own field collections. Of those records, 14,626 were identified specimens belonging to 373 species and morphospecies in around 220 genera and 49 families. The remaining 109 specimens were early juveniles or too damaged to be identified to the species level.

From the total of 353 species and morphospecies collected in our expeditions to the park, 195 were attributed to species already described (54.9%). Among the others, one genus and 72 species are considered as new to science (20.7%) and 86 were left in the level of morphospecies only (24.4%). The last category includes species represented only by juveniles or belonging to taxa without a proper taxonomic revision. Considering only the 14,492 specimens collected during the survey, 2,754 are males (19.0%) and 4,030 (27.8%) are females, with a total of 6,784 adults (46.8%). The remaining 7,708 (53.2%) are juveniles. Table 1 lists each recorded species, the stations in the park they were collected (S1, S2, S3 and S4), sex (M = male, F = female or J = juveniles) and total abundance (TAb) of specimens.

Among the 373 total species, 342 were represented by adult specimens and only 31 were represented by juveniles. Herein, 314 species were collected exclusively through the standardized survey (84.6% of the total species) with 287 represented by adults and only 29 represented by juveniles. The species represented only by juveniles were added to the database when it was clear that the spiders did not belong to any one of the other species already included. Almost all of the added species belonged to genera or even families not yet represented in the database.

A thorough analysis of the literature resulted in 17 papers containing records for 25 species and 37 specimens. Those records included specimens not only for the Park itself, but also to surrounding areas, as Jacarepaguá or only Pedra Branca. The database of Laboratório de Aracnologia, Museu Nacional/UFRJ, included 42 species/morphospecies and 83 specimens from the park and surrounding areas. The examination of the collection of the Laboratório de Diversidade de Aracnídeos/UFRJ revealed that 17 species and 26 specimens were from the same areas. The literature and collection records added up together 67 species/morphospecies and 134 specimens. Among the 12 species from the literature and that were not collected during the standardized survey, seven were labeled informing the Park itself or just “Pedra Branca”. Four species were cited only to a larger area that includes the Park (Jacarepaguá): *Teudis atrofasciatus*, *Xeropigo tridentiger*, *Peucetia flava*, *Peucetia rubrolineata* and one cited for Recreio dos Bandeirantes, an area nearby the Park: *Corinna demersa*. Moreover, among the eight species present only on collections, three were mentioned from the surroundings: *Actinosoma pentacanthum*, *Dendryphantes* sp. 01 and *Parasteatoda tepidariora*. The inclusion of those ten species in the list was made for the sake of completude and reflects our belief that they are probably present in the park area. They may be rare or inhabit areas not sampled by us.

### Species Abundance

**Figure 3.**
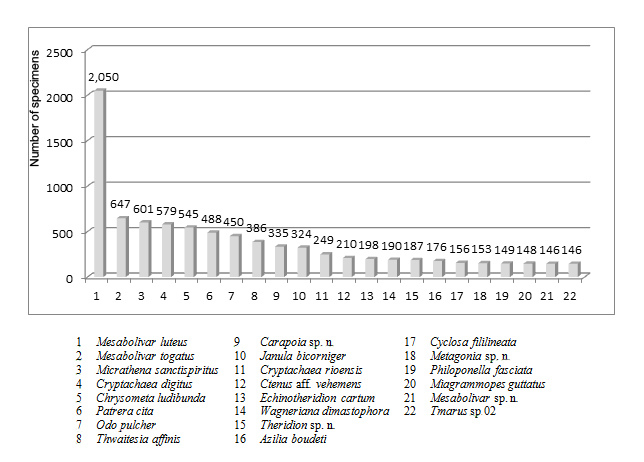
Abundance of collected species with more than 1% of total abundance.Suppl. material 1

Regarding species abundance, the 22 most abundant species (6.2% of total richness), represented by at least 1% of the total collected specimens, added up to 8,513 specimens (58.7% of total abundance) (Fig. 3). On the other hand, 74 species are represented by only one specimen (singletons) and 37 by two specimens (doubletons). These “rare species” represent a sizable piece of richness (33%), but only a small part of total abundance (1.1%). The ten most abundant species in order are: *Mesabolivar luteus* (2,050 specimens); *Mesabolivar togatus* (647); *Micrathena sanctispiritus* (601); *Cryptachaea digitus* (579); *Chrysometa ludibunda* (545); *Patrera cita* (488); *Odo pulcher* (450); *Thwaitesia affinis* (386); *Carapoia* sp. n. 02 (335) and *Janula bicorniger* (324).

The difference in abundance between *Mesabolivar luteus* (2,050 specimens) and the second most abundant species, *Mesabolivar togatus* (647 specimens) is clear-cut, where both are dominant species in the local spider fauna. Apparently, according to our field observations on these very dominant species, they do not compete against each other, as *M. luteus* occupies higher places in the vegetation in comparison to *M. togatus*. It is also noteworthy that other three species among the 22 more abundant species are Pholcidae, *Carapoia* sp. n. 02, *Mesabolivar* sp. n. and *Metagonia* sp. n.

In relation to family richness and abundance, our results were in line with similar surveys on Atlantic Forest. Theridiidae is the richest family with 66 species (18.7%) in 27 genera. On the other hand, its abundance is the second highest, with 3,160 specimens (21.6%). As the second richest family, we observed Araneidae with 47 species (13.3%) in 21 genera. This family is the third most abundant (2,005 specimens). The third richest family was Salticidae with 45 species (12.8%), in 27 genera, and 640 specimens (only 4.4% of the total). Pholcidae was the most abundant family, with 3,810 specimens (26.3% of the total), but the fifth richest one, with 17 species (4.8%). The relatively high richness of Pholcidae in Pedra Branca State Park represents the highest number of species for the family in the world. The previous record was 15 species of Pholcidae in Reserva Ecológica de Guapiaçu, Cachoeiras de Macacu, also in Rio de Janeiro state, Brazil (Huber and Rheims 2011).

### Richness estimates

To estimate the possible reach of the spider fauna, an accumulation curve was calculated using the most used estimators from literature by the software EstimateS Richness Estimator Program, Version 9.1 (Colwell 1999). Only data from the standardized surveys of the park were included in our analysis.

**Figure 4.**
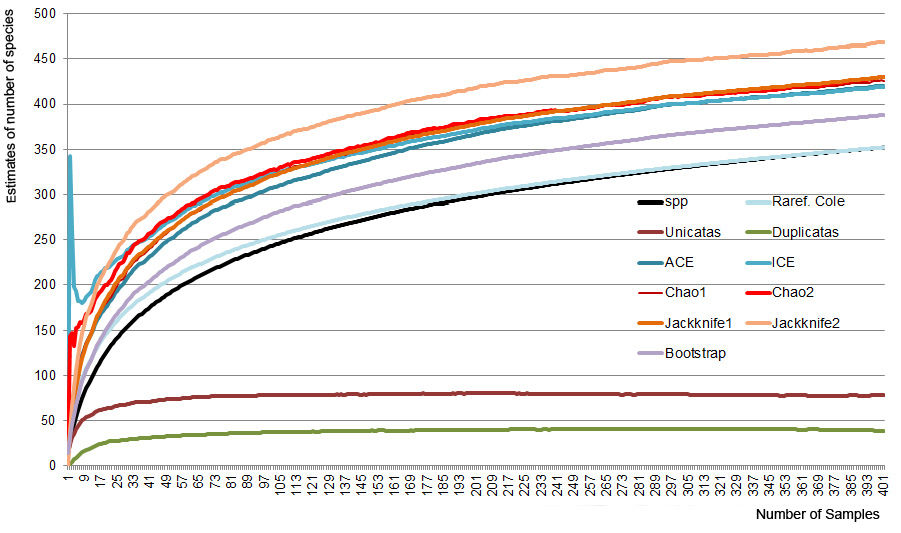
Species accumulation curve for different estimators of diversity, calculated by the software EstimateS v9.1, based only in standardized sampling. X axis: number of samples. Y axis: estimates of number of species Suppl. material 2).

Different estimators indicate a species total ranging from a minimum of 388 (“Bootstrap”) to a maximum of 468 species (“Jackknife2”) in this specific situation (Table 2, Fig. 4). Bootstrap estimator does not use only rare species to estimate the total richness, but all the samples obtained during the survey. It is calculated by adding up the total richness to the sum of the inverse proportion of samples in which every species occur (Smith and van Belle 1984). On the other hand, Jackknife is a general statistical technique for reducing the bias of an estimator by removing subsets of the data and recalculating the estimator. Jackknife2 adds the total observed richness to a parameter calculated from the number of individuals and of rare species found only in one (uniques) or two samples (duplicates) in order to obtain the total species richness (Gotelli and Colwell 2010).

In the present work, the species accumulation curves still have not reached an asymptote, but the curves slopes are apparently beginning to decrease. This may indicate that the curves are converging to a plateau and to stabilization on the estimated number of species. The effective number of species (353) is still lower than the lesser optimistic estimator (“Bootstrap”). Besides, the number of uniques reaches 78 and is exactly the double of the duplicates and the intersection between the two curves would only be reached by surveying the area during a few more years, which indicates the need for a higher collection effort.

### Diversity patterns

The analysis of diversity patterns of the spider community in the study area includes the comparison of population parameters for each sampling station individually or by each Park core, Pau da Fome (S1 & S2) and Camorim (S3 & S4). The diversity indexes used were Shannon-Wiener (H'), equitability of Pielou (J) and dominance of Berger-Parker (d) (Table 3).

In relation to alpha-diversity, H' was higher for Camorim (4.411), with an effective number (Shannon Exponential) of approximately 82 species. On the other hand, for Pau da Fome, H' was 4.051, with Shannon Exp of around 57 species. Individually, station 4 ("Açude") presented the highest rate for H' (4.331), with Shannon Exp of 76 species, followed by station 1, with H' of 3.999. These numbers indicate that Camorim core (stations 3 and 4) is more diverse than Pau da Fome (stations 1 and 2).

The high H' for station 4 is coupled to the highest equitability (J = 0.739) and the lowest dominance of one species (d = 0.055). In contrast to the dominance of *Mesabolivar luteus* in stations 1, 2 and 3, the most abundant species in Station 4 is *Chrysometa ludibunda*, with 204 specimens, whereas the second one is *Mesabolivar togatus* with 196 specimens. Those results, allied to the highest abundance and richness, indicates that station 4 is the best preserved and has the most complex environment among all stations.

### Species new records and distributions

According to the World Spider Catalog 2015, this survey also highlights some new distribution records for different areas: **Liocranidae** - new family distribution record for Brazil; **Tetragnathidae:** *Tetragnatha mandibulata* Walckenaer, 1841 - new species distribution record for Americas; **Linyphiidae:** *Erigone autumnalis* Emerton, 1882 - new species distribution record for South America; **Mysmenidae:** *Maymena* sp. n. – new genus record for Brazil; **Salticidae:** *Encolpius guaraniticus* Galiano, 1968 – new species record for Brazil and new genus record for Southeastern region; and **Tetragnathidae**: *Leucauge pulcherrima* (Keyserling, 1864) and **Theridiidae:** *Dipoena cornuta* Chickering, 1943, *Dipoena bryantae* Chickering, 1943 - all new species records for Brazil.

## Discussion and Conclusions

Our study uses standardized techniques alongside statistical tools to estimate the spider fauna in forested areas in Rio de Janeiro state. About Brazil as a whole, we can find studies documenting the fauna of spiders presenting a simple list of species (e. g. Buckup et al. 2010, Brescovit et al. 2011, Chavari et al. 2014, Melo et al. 2014, Nogueira et al. 2014), and others that consider statistical tools to analyze the dynamics of the fauna (e. g. Álvares et al. 2014, Indicatti et al. 2005, Candiani et al. 2005, Raizer et al. 2005, Nogueira et al. 2006).

In the state of Rio de Janeiro, the Laboratório de Diversidade de Aracnídeos is an active group working with spiders surveys in many different areas, like an ongoing work in the municipality of Macaé and other in the municipality of Mendes, which composed the monograph of Prado (2015). In the city of Rio de Janeiro, however, our knowledge is still very incipient, with information from Parque Municipal do Marapendi (Marapendi Municipal Park), which until now was the only area of the city entirely surveyed with records of 159 species (Baptista et al. 2015). Parque Nacional da Tijuca (Tijuca National Park) however, has already been partially surveyed as part of Rapid Ecological Survey during the elaboration of a new management plan for Tijuca Park. This study was included in the monograph of Silva-Moreira (2006), where 308 species of spiders are mentioned for Tijuca National Park. Also, before this survey at Pedra Branca State Park, in the city of Rio de Janeiro, there have never been any statistical treatments about the spider fauna diversity.

Furthermore, sampling efforts must be considered in each survey because the spider fauna recorded for Tijuca is the result of a Rapid Ecological Survey with only one expedition with standardized methods. However, this specific area has many sporadic records since the 19th century. Therefore, a beta diversity comparison between the fauna of Pedra Branca and Tijuca is still not feasible, but it is expected that they may share most of the spider species.

The survey in Pedra Branca overcame our initial expectations on species richness, especially considering that this Park is under high anthropic pressure. It was expected that areas under these conditions would only present a higher number of species of broad distribution, which may allow them to survive the human influence and to withstand a higher variation in environment factors. The remarkable richness recorded for this urban forest, even higher than in Tijuca, may be related to its location at the western portion of the city of Rio de Janeiro, an area where human occupation started later. Another reason may be the predominance of steep hillside areas, which makes it difficult to access protected areas of the park.

Moreover, the little number of cosmopolitan and pantropical species and the large number of Brazilian species, especially the ones restricted to the Southeastern region, may indicate that the area of Pedra Branca State Park is still well preserved. However, the low comparative data from other areas hinders any inference on the subject at the present moment. So, we conclude this work acknowledging that even areas with high anthropic pressure can provide important information in order to ensure the protection of what remains of this historically vast area.

## Supplementary Material

Supplementary material 1 Most Abundant Species at Pedra Branca State Park Data type: Statistical
Brief description: This is a comparison summary of the most abundant species at Pedra Branca State Park. This data was obtained from Table 1.
File: oo_61128.xlsx Castanheira, Pérez-González & Baptista

Supplementary material 2 Estimates Data type: Statistical
Brief description: This is the raw data that made possible the construction of the graphic that shows the estimates of species.
File: oo_61126.xlsx Castanheira, Pérez-González & Baptista

## Figures and Tables

**Table 1.** List of species recorded from Pedra Branca State Park.

**Species**	**S1**	**S2**	**S3**	**S4**	**M**	**F**	**J**	**TAb**
Amaurobiidae						5		5
*Retiro lanceolatus* (Vellard, 1924)		X				5		5
Anapidae					2	2		4
*Anapis* sp. n.		X			2	1		3
*Pseudanapis* sp. n.			X			1		1
Anyphaenidae					68	79	503	650
Anyphaenoides cf. clavipes (Mello-Leitão, 1922)				X			1	1
*Arachosia praesignis* (Keyserling, 1891)				X	1			1
*Aysha affinis* (Blackwall, 1862)			X	X		1	19	20
*Aysha borgmeyeri* (Mello-Leitão, 1926)		X	X	X	6	14	41	61
*Iguarima censoria* (Keyserling, 1891)				X		1		1
*Isigonia* sp. n.			X			1		1
*Jessica osoriana* (Mello-Leitão, 1922)				X		2		2
*Osoriella domingos* Brescovit, 1998					1			1
*Oxysoma* sp. 01	X						1	1
*Patrera cita* (Keyserling, 1891)	X	X	X	X	59	56	373	488
*Teudis angusticeps* (Keyserling, 1891)					1			1
*Teudis atrofasciatus* Mello-Leitão, 1929						1		1
Wulfilopsis cf. frenata (Keyserling, 1891)	X	X	X	X		1	68	69
*Xiruana* sp. 01			X			2		2
Araneidae					168	393	1484	2048
*Acacesia hamata* (Hentz, 1847)						1		1
*Acacesia tenella* (L. Koch, 1871)	X	X			1	1	2	4
*Actinosoma pentacanthum* (Walckenaer, 1841)					1			1
Alpaida aff. morro	X	X		X		6	7	13
*Alpaida alticeps* (Keyserling, 1879)		X		X		1	9	10
*Alpaida atomaria* (Simon, 1895)	X	X	X	X	4	9	15	28
*Alpaida tijuca* Levi, 1988	X	X	X	X	6	14	76	96
*Alpaida truncata* (Keyserling, 1865)	X	X	X	X	5	9	48	62
*Alpaida venger* Castanheira & Baptista, 2015	X		X	X	6	11	36	53
*Alpaida* sp. n.		X				1		1
*Araneus iguacu* Levi, 1991	X	X	X	X	5	29	21	55
*Araneus lathyrinus* (Holmberg, 1875)						1		1
*Araneus omnicolor* (Keyserling, 1893)	X	X	X	X	1	4	15	20
*Araneus stabilis* (Keyserling, 1892)	X	X	X	X	1	2	12	15
*Araneus tijuca* Levi, 1991	X	X	X	X	1	2	14	17
*Araneus venator* (C. L. Koch, 1838)	X	X	X	X		11	130	141
*Araneus* sp. 01	X						1	1
*Argiope argentata* (Fabricius, 1775)								1
*Cyclosa caroli* (Hentz, 1850)			X	X		2		2
*Cyclosa fililineata* Hingston, 1932	X	X	X	X	11	62	83	156
*Cyclosa morretes* Levi, 1999	X	X	X	X	6	14	23	43
*Eustala levii* Poeta, Marques & Buckup, 2010			X	X	1		2	3
Eustala aff. levii	X	X	X	X		1	10	11
Eustala aff. photographica	X	X	X	X	3	4	25	32
*Eustala sagana* (Keyserling, 1893)	X		X			3	12	15
*Eustala taquara* (Keyserling, 1892)		X				1	1	2
*Gasteracantha cancriformis* (Linnaeus, 1758)			X	X	1	1	1	3
*Kaira altiventer* O. Pickard-Cambridge, 1889				X			1	1
*Kapogea* sp. n.	X				1			1
*Mangora aripeba* Levi, 2007			X		6	9	3	18
*Mangora enseada* Levi, 2007			X			2		2
*Mangora melanocephala* (Taczanowski, 1874)					1	2		3
*Mangora missa* Levi, 2007	X	X			1	7	15	23
*Mangora ramirezi* Levi, 2007	X	X	X		4	12	10	26
*Metazygia bahia* Levi, 1995		X				1		1
*Metazygia laticeps* (O. Pickard-Cambridge, 1889)			X		1	2	5	8
*Metepeira* sp. 01				X			1	1
*Micrathena annulata* Reimoser, 1917	X	X	X	X	13	26	42	81
*Micrathena horrida* (Taczanowski, 1873)	X		X		2		4	6
*Micrathena jundiai* Levi, 1985					1			1
*Micrathena sanctispiritus* Brignoli, 1983	X	X	X	X	46	73	484	603
*Ocrepeira gnomo* (Mello-Leitão, 1943)		X		X		1	45	46
*Parawixia audax* (Blackwall, 1863)	X	X	X	X	1	8	32	41
*Parawixia monticola* (Keyserling, 1892)	X	X	X	X	5	1	15	21
*Parawixia velutina* (Taczanowski, 1878)			X	X		1	13	14
*Pronous tuberculifer* Keyserling, 1881		X	X	X	2	3	12	17
*Scoloderus cordatus* (Taczanowski, 1879)	X	X			4	5	5	14
*Taczanowskia striata* Keyserling, 1879		X					1	1
*Tatepeira itu* Levi, 1995				X	2	1		3
*Testudinaria* sp. n.	X	X	X		1	2	4	7
*Verrucosa meridionalis* (Keyserling, 1892)	X	X	X	X	1	1	33	37
*Wagneriana dimastophora* (Mello-Leitão, 1940)	X	X	X	X	13	33	145	191
*Wagneriana gavensis* (Camargo, 1950)	X	X	X	X	10	13	71	94
Barychelidae					1	1	3	5
*Neodiplothele fluminensis* Mello-Leitão, 1924				X		1	3	4
*Paracenobiobelma* sp. 01		X			1			1
Caponiidae					3	3	2	8
*Caponina tijuca* Platnick, 1994		X			2	2	1	5
*Nops* sp. n.		X		X	1	1	1	3
Clubionidae					2	5	7	14
Clubionidae sp. 01	X	X	X	X	2	5	7	14
Corinnidae					84	42	135	261
*Castianeira brevis* Keyserling, 1891								
*Castianeira* sp. 01	X	X			1	1		2
*Castianeira* sp. 02		X	X	X			4	4
Corinna aff. capito			X	X	3	1		4
*Corinna demersa* Rodrigues & Bonaldo, 2014						2		2
*Corinna inermis* (Bertkau, 1880)				X	1	1		2
Corinna aff. mourai				X	1	2	1	4
*Corinna nitens* (Keyserling, 1891)		X	X	X	4		12	16
*Corinna* sp. 01	X	X	X	X	1		5	6
*Corinna* sp. 02			X				1	1
*Corinna* sp. 03	X			X			2	2
*Creugas* sp. 01	X	X	X	X	14	9	23	46
*Creugas* sp. 02	X	X	X	X	34	14	11	59
*Ianduba varia* (Keyserling, 1891)	X		X	X	12	7	13	32
*Myrmecium obscurum* Keyserling, 1891			X	X	2		9	11
*Myrmecium rufum* Latreille, 1824			X	X	7	3	43	53
*Paradiestus* sp. 01				X			1	1
*Stethorrhagus* sp. n.			X			1		1
*Tupirinna* sp. n.		X	X	X	3		7	10
*Xeropigo tridentiger* (O. Pickard-Cambridge, 1869)					1			1
*Xeropigo* sp. n.				X		1		1
Ctenidae					81	66	343	490
*Ctenus medius* Keyserling, 1891	X	X	X	X	1	6	81	88
*Ctenus ornatus* (Keyserling, 1877)	X	X	X	X	8	13	88	109
Ctenus aff. vehemens	X	X	X	X	59	44	107	210
*Enoploctenus cyclothorax* (Bertkau, 1880)		X	X	X	6	2	26	34
Enoploctenus cf. maculipes Strand, 1909		X	X	X	2		28	30
*Isoctenus griseolus* (Mello-Leitão, 1936)	X	X		X	1		13	14
*Phoneutria keyserlingi* (F. O. Pickard-Cambridge, 1897)		X	X		4	1		5
Deinopidae						5	3	8
*Deinopis plurituberculata* Mello-Leitão, 1925			X	X		5	3	8
Dipluridae					3	3	6	12
*Diplura lineata* (Lucas, 1857)				X		1		1
*Linothele* sp. n.		X	X	X	3	2	6	11
Eutichuridae					5	3	8	16
*Radulphius laticeps* Keyserling, 1891		X		X	2		1	3
*Radulphius* sp. n.		X	X	X	2	3	6	11
*Strotarchus tropicus* (Mello-Leitão, 1917)				X	1		1	2
Gnaphosidae					1	2	6	9
*Apodrassodes* sp. n.		X				1		1
Poecilochroa cf. trifasciata Mello-Leitão, 1918	X						1	1
*Xenoplectus* sp. n.		X			1	1		2
*Zimiromus* sp. 01		X		X			5	5
Hahniidae					3	8	1	12
*Hahnia* sp. 01				X	3	7	1	11
*Neohahnia* sp. 01			X			1		1
Hersiliidae					4	2	3	9
*Ypypuera crucifera* (Vellard, 1924)	X		X	X	4	2	3	9
Idiopidae					5		1	6
*Idiops camelus* (Mello-Leitão, 1937)	X	X			4		1	5
*Idiops germaini* Simon, 1892		X			1			1
Linyphiidae					118	226	122	466
Dubiaranea cf. inquilina (Millidge, 1985)	X	X			11	52	13	76
*Erigone autumnalis* Emerton, 1882			X			1		1
*Exocora phoenix* Lemos & Brescovit, 2013	X	X	X	X	9	40	24	73
*Laminacauda* sp. n.				X		2	1	3
*Lygarina* sp. n.			X	X	2			2
Meioneta aff. montivaga				X	1			1
*Meioneta* sp. n. 01	X	X	X	X	27	37	27	91
*Meioneta* sp. n. 02			X		1	1	1	3
*Moyosi* sp. n.	X	X	X	X	30	33	23	86
*Sphecozone rubescens* O. Pickard-Cambridge, 1870	X					1		1
*Sphecozone* sp. n.	X					1	3	4
*Vesicapalpus simplex* Millidge, 1991	X	X	X	X	30	40	22	92
Erigoninae sp. 01	X		X		7	17	8	32
Erigoninae sp. 02			X			1		1
Liocranidae					12	9	8	29
Liocranidae sp. 01	X	X	X	X	12	9	8	29
Lycosidae						1	1	2
*Hogna* sp. 01						1	1	2
Mimetidae					44	46	125	215
*Gelanor altithorax* Keyserling, 1893		X	X	X	4	15	48	67
*Gelanor zonatus* (C. L. Koch, 1845)		X	X	X	9	8	14	31
*Mimetus* sp. 01	X	X	X	X	14	7	44	65
*Mimetus* sp. 02	X	X	X	X	11	9	10	30
*Mimetus* sp. 03	X	X	X	X	4	5	7	16
*Mimetus* sp. 04	X				1	1	2	4
*Mimetus* sp. 05		X			1	1		2
Miturgidae					188	149	129	466
*Odo pulcher* Keyserling, 1891	X	X	X	X	188	149	129	466
Mysmenidae					10	7	6	23
*Maymena* sp. n.	X	X	X		2	4	2	8
*Mysmena* sp. 01	X				1		1	2
*Mysmenopsis archeri* Platnick & Shadab, 1978	X	X	X		7	3	3	13
Nemesiidae					30	19	31	80
*Chaco* sp. n. aff.		X		X	3	7	2	12
Gen. n. sp. n.	X	X	X	X	10	2	10	22
*Prorachias* sp. n.	X				5	1	4	10
*Rachias conspersus* (Walckenaer, 1837)				X	4	2	5	11
*Rachias* sp. n.				X	2	1		3
*Stenoterommata melloleitaoi* Guadanucci & Indicatti, 2004			X	X	2	2		4
*Stenoterommata* sp. 01		X	X	X	2	4	9	15
*Stenoterommata* sp. 02		X		X	2		1	3
Nephilidae					2	11	29	42
*Nephila clavipes* (Linnaeus, 1767)	X	X	X	X	2	11	29	42
Ochyroceratidae					6	71	12	89
*Ochyrocera* sp. n. 01	X	X	X	X	5	14	6	25
*Ochyrocera* sp. n. 02				X	1			1
*Theotima minutissima* (Petrunkevitch, 1929)	X	X	X	X		57	6	63
Oonopidae					48	79	17	144
*Brignolia* sp. n.	X	X			3	4		7
*Neotrops* sp. n. 01	X	X	X	X	3	1	2	6
*Neotrops* sp. n. 02			X	X	3	4	4	11
*Neotrops* sp. n. 03	X	X	X	X	4	1	1	6
*Neoxyphinus keyserlingi* (Simon, 1907)	X	X	X	X	5	6	4	15
*Orchestina* sp. 01				X	1	3	1	5
*Triaeris stenaspis* Simon, 1891		X	X	X		5		5
Gamasomorphinae sp. 01	X	X	X	X	19	38		57
Gamasomorphinae sp. 02		X				1		1
Oonopinae sp. 01	X	X	X	X	10	14	4	28
Oonopinae sp. 02				X		1		1
Oonopinae sp. 03				X			1	1
Oonopinae sp. 04			X			1		1
Oxyopidae					1	2	3	6
Oxyopes cf. rubrosignatus Keyserling, 1891	X						1	1
*Peucetia flava* Keyserling, 1877						1		1
*Peucetia rubrolineata* Keyserling, 1877						1		1
*Schaenicoscelis elegans* Simon, 1898	X				1		2	3
Palpimanidae					4	5	7	16
*Fernandezina tijuca* Ramírez & Grismado, 1996	X	X	X	X	1	3	7	11
*Otiothops* sp. n.				X	3	2		5
Philodromidae							6	6
Berlandiella cf. insignis Mello-Leitão, 1929				X			6	6
Pholcidae					932	1112	1776	3820
*Carapoia* sp. n. 01	X	X		X	7	2		9
*Carapoia* sp. n. 02	X	X	X	X	87	132	116	335
*Litoporus iguassuensis* Mello-Leitão, 1918	X	X			16	36	40	92
*Mesabolivar brasiliensis* (Moenkhaus, 1898)			X		1	1		2
*Mesabolivar cyaneotaeniatus* (Keyserling, 1891)	X	X	X	X	20	17	71	108
*Mesabolivar difficilis* (Mello-Leitão, 1918)	X	X	X	X	18	38	33	89
*Mesabolivar luteus* (Keyserling, 1891)	X	X	X	X	494	539	1017	2050
*Mesabolivar togatus* (Keyserling, 1891)	X	X	X	X	143	143	362	648
*Mesabolivar* sp. n. 01	X	X	X	X	52	50	44	146
*Mesabolivar* sp. n. 02	X				1			1
*Mesabolivar* sp. n. 03			X			3	2	5
*Metagonia furcata* Huber, 2000				X		1		1
*Metagonia* sp. n. 01	X	X	X	X	32	83	38	153
*Metagonia* sp. n. 02			X			1	1	2
*Metagonia* sp. n. 03	X	X	X		37	41	30	108
*Metagonia* sp. n. 04				X	3	2		5
*Tupigea* sp. n. 01	X	X	X	X	21	23	22	66
Pisauridae					6	10	40	56
*Architis brasiliensis* (Mello-Leitão, 1940)				X	6	10	40	56
Prodidomidae						1		1
*Lygromma* sp. n. 01				X		1		1
Salticidae					121	125	399	645
*Acragas* sp. n.	X					1		1
*Arnoliseus* sp. n. 01		X	X	X	3	2		5
*Arnoliseus* sp. n. 02	X	X	X	X	18	14	23	55
Beata aff. zeteki		X				1		1
Breda cf. milvina (C. L. Koch, 1846)	X			X			2	2
*Chira lucina* Simon, 1902		X	X		1	1	2	4
*Chira thysbe* Simon, 1902		X	X			1	1	2
Chirothecia aff. semiornata		X			1			1
*Coryphasia albibarbis* Simon, 1902	X	X	X	X	14	3	124	141
Coryphasia aff. albibarbis			X			2		2
*Corythalia* sp. 01		X	X			1	3	4
*Cotinusa magna* (Peckham & Peckham, 1894)	X	X	X	X	3	4	25	32
*Cylistella cuprea* (Simon, 1864)		X	X	X	4	1		5
*Dendryphantes* sp. 01						1		1
*Encolpius guaraniticus* Galiano, 1968		X			1	3	1	5
*Erica eugenia* Peckham & Peckham, 1892	X	X	X	X	4	3	34	41
*Euophrys* sp. 01	X	X			2	1		3
*Freya* sp. n.			X		1			1
*Itata* sp. n.	X		X		1	1	4	6
*Lyssomanes austerus* Peckham, Peckham & Wheeler, 1889	X	X	X		3	3	10	16
Mago aff. longidens		X	X		2			2
*Mopiopia bruneti* Simon, 1903	X		X	X	1	2	5	8
*Mopiopia gounellei* Simon, 1902	X	X	X	X	2	3	10	15
*Mopiopia* sp. 01	X	X			1	3	3	7
*Noegus bidens* Simon, 1900	X	X	X	X	6	1	18	25
*Noegus comatulus* Simon, 1900	X	X	X	X	22	35	81	138
*Phiale mimica* (C. L. Koch, 1846)			X			1	1	2
Pseudofluda cf. pulcherrima Mello-Leitão, 1928				X		1		1
*Romitia* sp. 01		X					1	1
*Semnolius* sp. n.		X	X		1	1	6	8
*Semnolius* sp. 01	X	X	X	X	5	9	9	23
Tacuna aff. vaga	X	X		X	1	2	2	5
Tariona aff. mutica	X	X	X		2		7	9
*Thiodina* sp. n.			X			1		1
*Vinnius uncatus* Simon, 1902		X		X		1	2	3
Euophryinae sp. 01	X	X	X		1	2	2	5
Salticidae sp. 01	X		X		2	1	2	5
Salticidae sp. 02	X	X				1	2	3
Salticidae sp. 03	X	X		X	3	9	3	15
Salticidae sp. 04			X	X	10	1	11	22
Salticidae sp. 05	X			X	2	1		3
Salticidae sp. 06				X		2		2
Salticidae sp. 07			X	X		1	3	4
Salticidae sp. 08		X		X	1	1		2
Salticidae sp. 09				X		1		1
Salticidae sp. 10			X		3	2	2	7
Scytodidae					6	4	18	28
*Scytodes itapevi* Brescovit & Rheims, 2000		X	X	X	2		2	4
Scytodes aff. lineatipes	X	X	X	X	4	4	16	24
Segestriidae						2	2	4
Ariadna cf. obscura (Blackwall, 1858)			X	X		2	1	3
*Ariadna* sp. 01		X					1	1
Selenopidae					1	1		2
*Selenops melanurus* Mello-Leitão, 1923			X		1	1		2
Senoculidae							3	3
*Senoculus iricolor* (Simon, 1880)		X					1	1
*Senoculus* sp. 01				X			2	2
Sicariidae					6	13	16	35
*Loxosceles adelaida* Gertsch, 1967		X			6	13	16	35
Sparassidae					15	14	102	131
*Caayguara albus* (Mello-Leitão, 1918)		X		X	1		1	2
*Caayguara cupepemassu* Rheims, 2010					1			1
*Caayguara cupepemayri* Rheims, 2010		X	X	X	1	1	4	6
*Caayguara pinda* Rheims, 2010	X	X	X	X	12	12	95	119
*Polybetes rapidus* (Keyserling, 1880)	X			X		1	1	2
*Stasina americana* Simon, 1887		X					1	1
Synotaxidae					1	1	8	10
*Synotaxus longicaudatus* (Keyserling, 1891)	X	X	X		1	1	8	10
Tetragnathidae					117	213	495	825
*Azilia boudeti* Simon, 1895	X	X	X	X	7	20	149	176
*Chrysometa boraceia* Levi, 1986				X	1			1
*Chrysometa ludibunda* (Keyserling, 1893)	X	X	X	X	98	157	290	545
*Chrysometa* sp. n.			X			1		1
*Dolichognatha pinheiral* Brescovit & Cunha, 2001	X				2		3	5
*Leucauge formosa* (Blackwall, 1863)			X	X		4	7	11
*Leucauge pulcherrima* (Keyserling, 1865)			X			12	8	20
*Leucauge turbida* (Keyserling, 1893)	X		X			5	13	18
*Tetragnatha cladognatha* Bertkau, 1880			X		5	10	17	32
*Tetragnatha mandibulata* Walckenaer, 1841					1			1
*Tetragnatha* sp. 01			X		1	1		2
Metinae sp. 01	X			X	2	3	8	13
Theraphosidae					7	9	6	22
*Catumiri* sp. 01		X		X		2	3	5
*Eupalaestrus spinosissimus* Mello-Leitão, 1923			X			1		1
*Homoeomma familiare* Bertkau, 1880			X	X	1	1	1	3
*Lasiodora fallax* (Bertkau, 1880)				X		1		1
*Magulla buecherli* Indicatti *et al.*, 2008		X					1	1
*Plesiopelma* sp. 01		X	X	X	6	4	1	11
Theridiidae					577	1206	1377	3160
*Achaearanea tingo* Levi, 1963	X					1	2	3
*Anelosimus dubiosus* (Keyserling, 1891)				X		5	1	6
*Anelosimus ethicus* (Keyserling, 1884)	X					2		2
*Anelosimus studiosus* (Hentz, 1850)	X			X		1	1	2
*Argyrodes elevatus* Taczanowski, 1873	X			X	2	6	2	10
*Chrosiothes niteroi* Levi, 1964	X	X	X	X	16	31	15	62
*Chrosiothes* sp. n.		X		X	1	9	8	18
*Chrysso compressa* (Keyserling, 1884)	X	X	X	X	4	12	85	101
*Coleosoma floridanum* Banks, 1900			X			1	1	2
*Cryptachaea bellula* (Keyserling, 1891)	X					1		1
*Cryptachaea dea* (Buckup & Marques, 2006)	X	X	X	X	18	30	15	63
*Cryptachaea digitus* (Buckup & Marques, 2006)	X	X	X	X	32	162	385	579
*Cryptachaea hirta* (Taczanowski, 1873)	X			X	2	3		5
*Cryptachaea inops* (Levi, 1963)	X	X	X	X	1	8	6	15
*Cryptachaea passiva* (Keyserling, 1891)	X	X	X	X	8	45	29	82
Cryptachaea aff. pilaton			X			1	1	2
*Cryptachaea rioensis* (Levi, 1963)	X	X	X	X	42	175	32	249
*Cryptachaea sicki* (Levi, 1963)				X	1			1
*Cryptachaea triguttata* (Keyserling, 1891)	X	X	X	X	8	26	20	54
*Cryptachaea* sp. n. 01			X			1		1
*Cryptachaea* sp. 01				X			1	1
*Dipoena bryantae* Chickering, 1943			X			1		1
*Dipoena cornuta* Chickering, 1943	X			X	2	3	3	8
Dipoena aff. cordiformis		X			1			1
Dipoena aff. hortoni				X		1		1
*Dipoena ira* Levi, 1963	X	X	X	X	17	14	10	41
Dipoena aff. kuyuwini			X	X		2		2
*Dipoena militaris* Chickering, 1943	X	X	X		2	3	3	8
*Dipoena niteroi* Levi, 1963	X	X	X	X	23	21	11	55
*Dipoena pumicata* (Keyserling, 1886)	X	X	X	X	29	17	50	96
*Dipoena pusilla* (Keyserling, 1886)	X			X	2	2	2	6
*Dipoena variabilis* Levi, 1963			X	X	1	1	1	3
*Dipoena* sp. n. 01	X		X		3	1		4
*Dipoena* sp. n. 02				X		1		1
*Dipoena* sp. n. 03	X		X	X	1	6		7
*Echinotheridion cartum* Levi, 1963	X	X	X	X	11	94	93	198
*Neopisinus cognatus* (O. Pickard-Cambridge, 1893)			X	X	7	7	8	22
*Exalbidion* sp. n.	X	X	X		7	9	5	21
*Faiditus acuminatus* (Keyserling, 1891)	X		X		2	4	5	11
*Faiditus caudatus* (Taczanowski, 1874)	X	X	X	X	15	9	14	38
Faiditus aff. jamaicensis			X	X		3		3
*Janula bicorniger* (Simon, 1894)	X	X	X	X	98	89	137	324
Lasaeola aff. donaldi				X	1			1
*Neospintharus rioensis* (Exline & Levi, 1962)				X	1	1		2
*Parasteatoda tepidariora* (C. L. Koch, 1841)						1		1
*Parasteatoda tesselata* (Keyserling, 1884)			X		1			1
*Phoroncidia rubromaculata* (Keyserling, 1886)				X		3		3
*Platnickina mneon* (Bösenberg & Strand, 1906)							1	1
*Rhomphaea metaltissima* Soares & Camargo, 1948	X				2		2	4
*Spintharus gracilis* Keyserling, 1886	X	X	X	X	6	3	1	10
*Stemmops* sp. n. 01	X	X	X	X	11	12	3	26
*Stemmops* sp. n. 02	X	X	X	X	19	35	13	67
*Stemmops* sp. n. 03				X	1	2	1	4
*Styposis* sp. n.	X		X		18	22	7	47
*Theridion biezankoi* Levi, 1963	X	X	X		1	5	9	15
*Theridion calcynatum* Holmberg, 1876			X		3	3	4	10
Theridion aff. hispidum	X				1			1
*Theridion teresae* Levi, 1963	X	X	X	X	15	29	13	57
*Theridion* sp. n. 01	X		X	X	16	1		17
*Theridion* sp. n. 02	X	X	X	X	2	12	6	20
*Theridion* sp. n. 03			X	X	33	112	43	188
*Theridion* sp. n. 04	X	X			16	16	9	41
*Theridion* sp. n. 05	X	X	X	X	7	10		17
*Thwaitesia affinis* O. Pickard-Cambridge, 1882	X	X	X	X	38	47	301	386
*Thymoites* sp. n.	X	X	X		26	76	9	111
*Tidarren haemorrhoidale* (Bertkau, 1880)	X	X	X	X		3	9	12
*Wamba crispulus* (Simon, 1895)	X				1	5		6
*Wirada tijuca* Levi, 1967			X	X	2			2
Theridiosomatidae					15	25	13	53
*Chthonos tuberosa* (Keyserling, 1886)	X	X	X	X	7	10	5	22
*Chthonos* sp. n.			X	X	3	3	3	9
*Theridiosoma* sp. n. 01	X	X	X		4	12	2	18
*Theridiosoma* sp. n. 02	X		X		1		1	2
*Theridiosoma* sp. n. 03	X						1	1
Wendilgarda cf. nigra Keyserling, 1886				X			1	1
Thomisidae					38	24	245	307
Acentroscelus cf. secundus Mello-Leitão, 1929		X	X	X	2	5	13	20
*Epicadinus gavensis* Soares & Soares, 1946	X	X	X		2	1	3	6
*Epicadus planus* Mello-Leitão, 1932			X				2	2
Misumenops cf. callinurus Mello-Leitão, 1929			X			1		1
*Onocolus simoni* Mello-Leitão, 1915	X	X	X		3		9	12
*Strophius nigricans* Keyserling, 1880		X				1		1
Tmarus aff. albolineatus		X					1	1
*Tmarus atypicus* Mello-Leitão, 1929		X	X	X		1	2	3
*Tmarus* sp. n.	X	X		X	4		12	16
*Tmarus* sp. 01	X	X		X	9	3	49	61
*Tmarus* sp. 02	X	X	X	X	16	10	120	146
*Tmarus* sp. 03		X					2	2
*Tmarus* sp. 04	X	X	X	X	1	2	18	21
Tobias cf. caudatus Mello-Leitão, 1929	X		X	X	1		14	15
Trachelidae					9	7	15	31
*Trachelas robustus* Keyserling, 1891	X	X				2	3	5
*Trachelas vitiosus* Keyserling, 1891	X	X	X	X	9	5	12	26
Trechaleidae					1	2	34	37
Enna aff. redundans	X	X	X	X	1		24	25
*Trechalea bucculenta* (Simon, 1898)						2		2
*Trechaleoides biocellata* (Mello-Leitão, 1926)		X	X				10	10
Uloboridae					39	77	187	303
*Miagrammopes guttatus* Mello-Leitão, 1937	X	X	X	X	13	19	116	148
*Philoponella fasciata* (Mello-Leitão, 1917)	X	X	X	X	25	55	70	150
*Philoponella vittata* (Keyserling, 1881)			X			2		2
*Uloborus* sp. 01	X	X			1	1	1	3
Zodariidae					1	4	7	12
*Tenedos eduardoi* (Mello-Leitão, 1925)	X			X	1	4	7	12
**Total**					**2,785**	**4,094**	**7,744**	**14,626**

**Table 2.** Results of non paramethric estimators of richness, number of collected species, unicates and duplicates for the data obtained by standardized sampling in Pedra Branca State Park.

**Species**	353	**Chao1**	425.09
**Uniques**	78	**Chao 2**	429.81
**Duplicates**	39	**Jackknife1**	429.81
**ACE**	420.12	**Jackknife2**	468.71
**ICE**	419.08	**Bootstrap**	387.89

**Table 3.** Diversity indexes of the spider community of Pedra Branca State Park.

Locality	Richness	Shannon (H’)	Shannon Exp	Pielou (J)	Berger-Parker (d)
Pau Fome	**248**	**4.051**	**57.455**	**0.691**	**0.189**
Station 1	182	3.999	54.544	0.682	0.131
Station 2	199	3.784	43.992	0.645	0.243
Camorim	**291**	**4.411**	**82.352**	**0.752**	**0.091**
Station 3	216	3.960	52.457	0.675	0.158
Station 4	222	4.331	76.020	0.739	0.055
